# Hybrid Areas of Work Between Employment and Self-Employment: Emerging Challenges and Future Research Directions

**DOI:** 10.3389/fsoc.2019.00086

**Published:** 2020-01-21

**Authors:** Annalisa Murgia, Rossella Bozzon, Pierluigi Digennaro, Petr Mezihorak, Mathilde Mondon-Navazo, Paolo Borghi

**Affiliations:** Department of Social and Political Sciences, University of Milan, Milan, Italy

**Keywords:** hybridity, solo self-employment, comparative research, cross-national ethnography, labor laws, collective forms of representation

## Abstract

The growth of non-standard employment relations has created one of the major challenges in terms of workers' rights as well as collective representation in European societies. Among non-standard employment relations, so-called “solo self-employed”—self-employed workers without employees—are challenging the very foundations of our labor markets, that is to say the opposition between employers and employees, fostering the development of emerging “hybrid” areas of work. The heterogeneity of the solo self-employed is difficult to capture from official statistics, which are still based on traditional classifications, and questions also the legal categories that qualify these workers. Moreover, the fact that solo self-employed workers do not form a homogenous group, and are diverse in terms of their activities, interests and needs, calls for changes in the way trade unions, employer organizations, and new freelancer associations develop collective actions, claims-making activities, and strategies of organizing. With the aim to achieve an in-depth understanding of the increasingly extensive and populated categories of the solo self-employed, this contribution aims at reconstructing the state of the art within different fields of study, such as employment relations, labor law, industrial relations and social movements, and at offering some possible future research directions.

## Introduction

The growth of non-standard employment relations has created one of the major challenges in terms of workers' rights as well as collective representation in European societies (Cordova, 1986; Supiot et al., 1998). Among non-standard employment relations, the so-called “solo self-employment”—self-employed workers without employees or “own account workers”—is increasingly intertwined with precarious forms of work, in which individuals have low legal protection, a limited coverage in terms of social security provisions, little capacity for savings, insurance or pensions, and are hardly included in traditional interest representation (Stanworth and Stanworth, 1995; Schulze Buschoff and Schmidt, 2009; Dekker, 2010; Keune, 2013; Spasova et al., 2017; Jansen and Sluiter, 2019). On top on this, solo self-employment is a category that is challenging our understanding of the nature of employment relationship, that is to say the opposition between employers and employees, and is also encouraging discussion around the emergence of “hybrid” areas of work (Murgia et al., 2016; Armano and Murgia, 2017; Murgia and Pulignano, 2019).

The heterogeneity of the solo self-employed is difficult to capture from official statistics, which are still based on traditional classifications, and also questions the legal categories that that these workers qualify for (D'Amours and Crespo, 2004; Muehlberger and Pasqua, 2009; Cappelli and Keller, 2013; Cieślik, 2015; Bennaars, 2019). Moreover, the fact that solo self-employed workers do not form a homogenous group, and are diverse in terms of their activities, interests and needs, calls for changes in the way trade unions, employer organizations, and new freelancer associations develop collective actions, claims-making activities and strategies of organizing (Heery and Abbott, 2000; Pernicka, 2006; Gumbrell-McCormick, 2011; Wynn, 2015; Jansen, 2017). This contribution aims at introducing the main emerging challenges discussed within different fields of study, such as employment relations, labor law, industrial relations and social movements. In the conclusions, a future agenda for research is proposed, with the aim of contributing to the development of transdisciplinary and multi-method approaches, more able to grasp the emerging “hybrid areas of work” and achieve an in-depth understanding of the increasingly extensive and populated categories of the solo self-employed.

## Trends and Heterogeneity of Solo Self-Employment

The twentieth century was marked by a constant decline in self-employment in favor of an increase in salaried employment, mainly due to the rise of the “Fordist model” (OECD, 2000; Supiot, 2001). In recent decades, however, there has been a reversal of this trend and a progressive increase in the number of self-employed workers in Europe, particularly when looking at the self-employed without employees (or “own account workers”). At the macro level, three main drivers have been identified to explain this trend. Firstly, solo self-employment has been a response to the shift from the industrial to a service economy and to the deep (de)regulation processes that have affected all European countries, including the erosion of the social position of many workers and, in some cases, the increased levels of unemployment (see Arum and Müller, 2004). Secondly, there have been unprecedented changes connected to internationalization, new technologies and decentralization of production, with increasing outsourcing activities by enterprises (see Bologna, 2018). Finally, socio-cultural trends have played a crucial role too, mainly by promoting autonomy and the idea of becoming “entrepreneurs of themselves” (Foucault, 2008) as the model to aspire.

**Figure 1:**
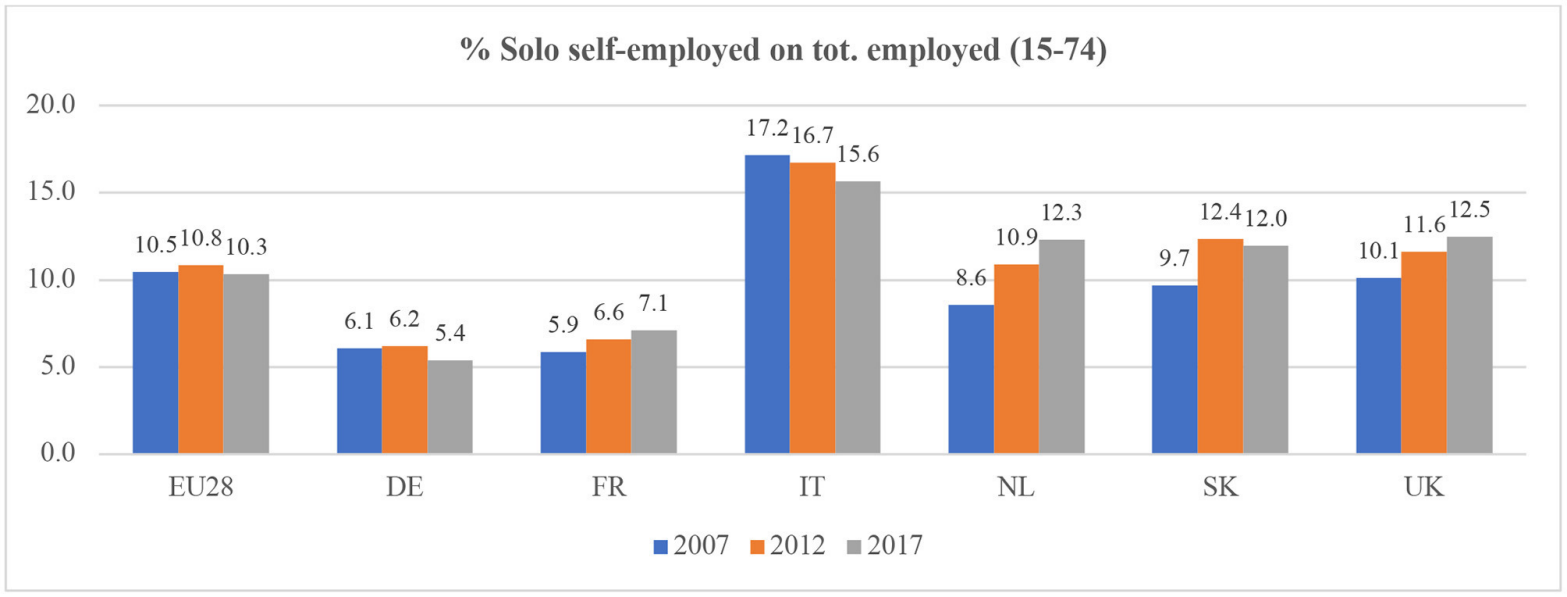
The development of self-employment in the EU and a number of selected European countries, 2007–2012–2017 (self-employment as a % of total employment). Source: Own calculations on Eurostat online database “Employment and unemployment (LFS)” [lfsa_esgais] https://ec.europa.eu/eurostat/data/database.

**Figure 2:**
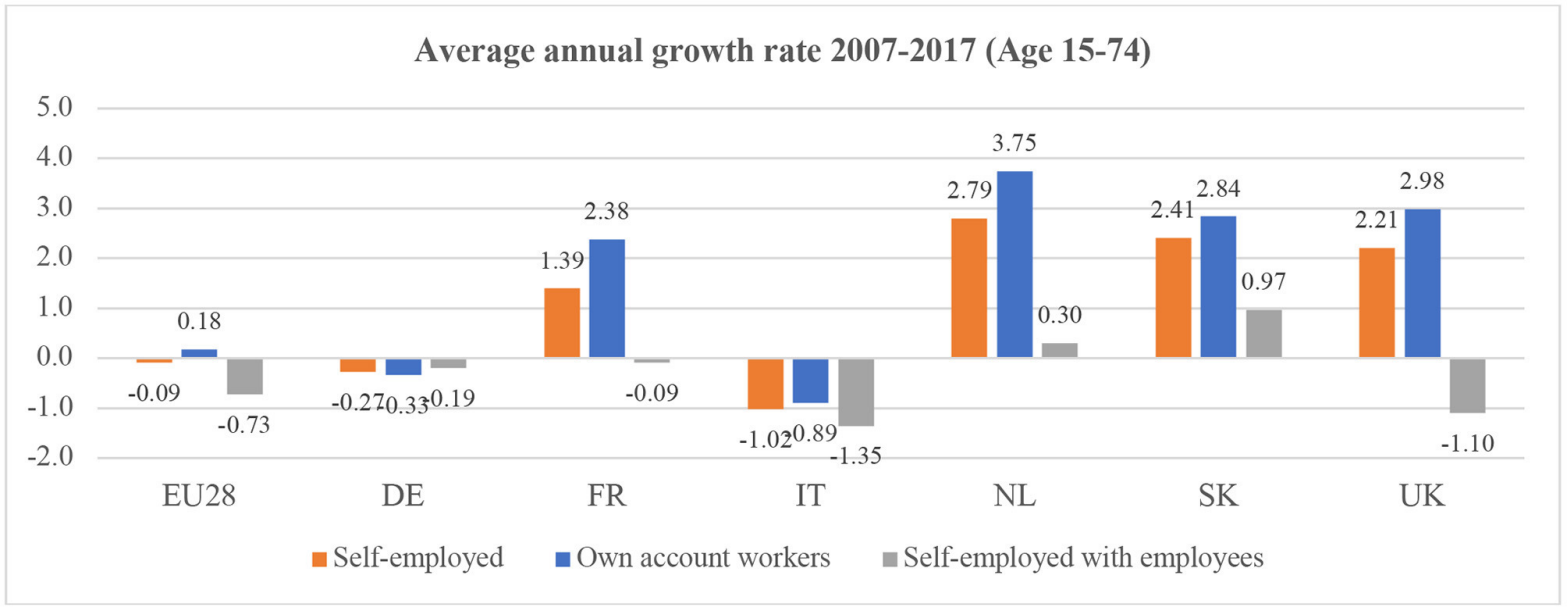
Average annual growth of self-employment in the EU and a number of selected European countries, 2007–2017. Source: Own calculations on Eurostat online database “Employment and unemployment (LFS)” [lfsa_esgais] https://ec.europa.eu/eurostat/data/database.

In this common frame, however, the heterogeneity of solo self-employed workers is extremely high. In terms of sectors, they can be found in areas with many high-skilled professionals as well as in low-skilled jobs: from civil engineering, journalism and ICT, to care homes, agriculture and construction (Eichhorst et al., 2013). As regards their composition, women are increasingly involved in these work arrangements, as well as young people and migrant workers (both among those starting micro-businesses and those hired on a solo self-employed contract because of a lack of other options, possibly related to their migrant status) (Mills and Blossfeld, 2005; Muehlberger and Pasqua, 2009; Galgóczi et al., 2012; Bozzon and Murgia, 2020). Moreover, the solo self-employed are variously distributed within the European Union (see Figures 1, 2). Indeed, in 2015, some countries had self-employment rates below 10% (8% in Denmark and 9% in Estonia and Luxembourg) and some countries had quite high rates, such as Greece (31%) and Italy (23%) (Eurofound, 2017).

Official statistics are then able to distinguish between self-employed workers with and without employees. In some cases, it is also possible to identify “dependent self-employed workers,” who do not have neither employees nor economic autonomy and control over their business (Eichhorst et al., 2013; Eurofound, 2017; Mondon-Navazo, 2017). However, beyond these classifications, statistics do not currently allow for consideration of the high heterogeneity of solo self-employment, where we can find genuine self-employment, but also a growing precariousness (including among workers who enjoy working as freelancers), as well as bogus or imposed false self-employment (Schulze Buschoff and Schmidt, 2009; Westerveld, 2012; Leighton, 2015; Borghi et al., 2018; Conen and Schippers, 2019). Moreover, these conditions may occur to the same person, and possibly even simultaneously, especially to those people who perform different jobs at the same time. Therefore, the increasingly blurred boundaries between self-employment and employment are challenging not only the indicators used by labor force surveys, but the very analytical categories used by academic scholars. The current debate is struggling to analyse these emerging hybrid areas of work, which are, in addition, differently regulated by welfare systems and labor laws at national level.

## Self-Employment in the Frame of Labor Law

As mentioned in the previous section, the concept of self-employment embraces a large variety of situations—such as bogus self-employment, economically dependent autonomous workers, platform workers, self-employed persons offering personal work or service to a multitude of clients/customers, small entrepreneurs and so on—that are also challenging the current juridical categories. Despite the described rapid changes in the few last decades, most of the national labor law systems still revolve around a dichotomy between subordinate/dependent employment, on the one hand, and autonomous/independent self-employment, on the other.

The self-employment concept is normally carved out in contrast with that of subordination. If one looks both at the statutory definitions of the concepts of employment, employee or contract of employment, as well as at the tests that courts have developed in many European countries, it is possible to realize that there is a common core of criteria that have been used to identify subordinate employment (Davidov et al., 2015; Countouris and De Stefano, 2019). The main criteria generally adopted, among others, can be brought back to three related macro-sets: hetero-direction of the work and its external control, which implies the power for the employer to give instructions and direct the employee's work; hetero-organization, which means that the performed work is integrated into someone else organization and business; and risk assessment, which essentially investigates whether the worker takes the ultimate risk of loss or chance of profit (Digennaro, 2019). Since the employment contract can also be described as a set of powers in favor of the employer, a different perspective assembles the criteria utilized in different legal traditions worldwide around the investigation into the presence of hierarchical power (which entails directional, control and disciplinary powers) (Casale, 2011).

Considering the above criteria, it is easy to observe how they match well with the “Taylor-Fordist model,” in which large companies were engaged in mass production on big factories, where the workforce was arranged according to a pyramidal-hierarchical organization. When the way of production changed and vertical integration was abandoned, many workers who performed tasks as employees in substance started to be formally engaged as self-employed or sub-contractors to reduce costs. Therefore, the new social reality has made it more complicated for normative systems to organize work organization through the category of subordination. The issues that this phenomenon raised are 2-fold. First, since the set of laws directed at protecting labor relied on the concept of subordination, the consequence of an extensive recourse to self-employment has been the exclusion from the domain of the employment protection legislation of broad classes of the active workforce (Collins, 1990). In many countries, the legislators' response has consisted in the extension, to varying degrees, of portions of labor and social rights to workers who are in a position of economic dependence or quasi-subordination by means of different techniques. Secondly, despite the similar position that the bogus and economically dependent self-employed share with subordinate employees, the former category does not always have access to the full enjoyment of trade union rights, and particularly to the right of collective bargaining, because of the competition law both at national and EU levels (Rubiano, 2013; De Stefano and Aloisi, 2018).

## The Collective Representation of Solo Self-Employed Workers

The progressive erosion of the standard employment relations has also prompted debate on the adequacy and effectiveness of structures and methods of collective representation. In the attempt to explore the substantial gap in union density between standard and non-standard workers, the current scientific debate is mainly focused on explaining national differences in unions' responses to the expansion of atypical jobs. Everywhere, unions have to deal with the emergence of a variety of atypical employment relationships—fixed-term, temporary agency and wage-limited part-time contracts, as well as solo self-employed positions—with low employment security and pay levels, which weaken the collective agreements and the minimum-wage bargained for dependent and permanent employees (Hyman, 1999; Heery and Abbott, 2000). However, in many countries, unions—whose members traditionally formed a homogeneous group of workers—struggle to deal with such fragmentation and different interests, and often continue to use their traditional strategies to curb temporary employment and tackle the precarious aspects of such contracts (Pernicka, 2005). It is especially in national contexts with strong legal employment protections that unions have belatedly developed bargaining capacities that addressed temporary workers. In more deregulated institutional regimes, like that of the UK, there are instead many examples of trade unions organizing in areas of casual or insecure employment, such as transport and construction, as well as the creative industries (Heery et al., 2004; Böheim and Muehlberger, 2006; Saundry et al., 2006; MacKenzie, 2009, 2010; Simms and Dean, 2015).

At present, despite the fact that most national trade unions have the right to recruit and organize self-employed workers, they are at the same time aware that they had not done enough for this category of workers in the past. Therefore, organizing and extending collective bargaining to the self-employed is now perceived as a priority across Europe (Fulton, 2018). Over the last 10 years, a number of scholars have studied the integration of the self-employed into union movements in Europe (Pernicka, 2006; Pedersini and Coletto, 2009; Gumbrell-McCormick, 2011; see Countouris and De Stefano, 2019), including the representation of platform workers, who are also part of the kaleidoscopic world of self-employment (Lenaerts et al., 2018; Vandaele, 2018). In general, there are differing views on the changing face of collective representation, and specifically on the future of collective bargaining for the solo self-employed (Keune, 2013). To use a standard categorization, they can be distinguished in the industrial unionism providing vertical integration of individuals in the same workplace regardless of occupation, and in the craft unionism providing horizontal extension by enlarging similar occupational groups instead. Also, in some countries, trade unions have even opposed the growth of solo self-employment, fearing that it would have undermined both standard employment relations and union solidarity (Goslinga and Sverke, 2003; Pernicka, 2006).

More recently, research has begun to investigate to what extent and under which conditions the solo self-employed are able to develop collective practices of organizing, focusing not only on unions, but also on chambers of commerce, business associations, cooperatives, new freelancers associations, and more grassroots claims-making activities (Murgia and Selmi, 2012; Battisti and Perry, 2015; Wynn, 2015; Brandl and Lehr, 2016; Hyman and Gumbrell-McCormick, 2017; Jansen, 2017; Bologna, 2018; Mezihorák et al., 2019; Murgia and de Heusch, 2020). In fact, given their heterogeneous composition, it is not surprising that this hybrid group of workers encounters difficulties in being represented by traditional systems of collective representation. The forms of organization through which self-employed workers mobilize are very different from one another, as is the range of what they are able to offer. For instance, collective bargaining is mainly carried out by unions and employer organizations, while other services can be offered by different types of association, such as legal and financial advice, work insurance, training, better access to social protection, involvement in collective consultation by government or local authorities, and new forms of mobilizing to improve working conditions.

## Our Proposal for a Future Research Agenda

Having critically discussed the main emerging challenges about the growing group of solo self-employed workers in different fields of study, this contribution aims at participating in this articulated debate by proposing a future research agenda able to allow a more fine-grained analysis of the heterogeneous category of solo self-employment. With this in mind, a transdisciplinary and multi-method original research approach is discussed, through which to study the “hybrid areas of work” and their impacts on national labor force surveys, labor laws and collective forms of representation.

From a theoretical perspective, the research on solo self-employment is fragmented into different fields of study and methodological approaches, which rarely open conversations to discussions from different disciplinary and epistemological angles. Many studies have been conducted with a quantitative causal-comparative approach, focused on the impact of these forms of employment on the enjoyment of workers' rights, social protection, and collective representation (e.g., Arum and Müller, 2004; Eichhorst et al., 2013). Other authors have instead explored the same phenomenon focusing on the meanings that solo self-employed workers attribute to their positions in the labor market (e.g., Barley and Kunda, 2004; Osnowitz, 2010; Armano and Murgia, 2017). This non-communicability of perspectives has proved to be an opportunity to reflect on the significance of engaging with different approaches and fields of study.

In studying solo self-employment, a promising future research pathway could be paved by opening a conversation between labor law, employment and industrial relations, and social movements studies, therefore fostering a “transdisciplinary approach” to the study of the hybrid areas of work. Differently from the idea of interdisciplinarity, where diverse disciplines are combined and integrated, along with their methodologies and assumptions, transdisciplinarity defines research focused on problems that cross disciplinary boundaries, aiming at a holistic approach and at a unity of knowledge (Arthur et al., 1989; Zaman and Goschin, 2010). In particular, a “subject-oriented” perspective (Beck and Beck-Gernsheim, 1996; Armano and Murgia, 2013) could be particularly appropriate for pursuing this objective. This means to systematically take into account reciprocal impacts between subjects and social structures, keeping together a micro- and a macro level analysis, in order to understand how subjects are affected by social norms and institutions, but also how they can shape them in turn. In the attempt to adopt this perspective, and to better understand the consequences of solo self-employment for social and legal protection and collective representation, different levels of analysis need to be addressed at the same time:

- How national and European statistics illustrate the world of self-employment, focusing on how data are differently collected through surveys on the labor force and whether they allow for the understanding of how the world of work is evolving;
- How the figure of the solo self-employed is regulated in labor laws at national and supranational levels, taking into account both the individual and the collective dimensions;
- How forms of collective representation are emerging, focusing both on the more institutionalized collective actors, such as unions and employer organizations, and the more fluid and new associations, cooperatives, grassroots groups, and forms of social movement unionism.

From a methodological perspective, pursuing this research agenda requires the use of a “multi-method research design” (Morse, 2003), which means that different methods are used in the same project, each conducted rigorously and complete in itself, and then used together to form essential components of a single research programme. In particular, multi-sited and cross-national ethnographies (Marcus, 1995; Mangen, 1999) can be particularly suited to the exploration of an emergent and transnational process—as it is the case of the emergence of “hybrid areas of work”—because of its capacity to combine interpretative “thickness” with comparability among different national contexts. Moreover, the “ethnography of contemporary worlds” is considered to be a multiple method on both a theoretical and technical level. In terms of research techniques, participant observation has become one among several tools of ethnography, which cannot renounce the analysis of documents, the reconstruction of the legal framework, the use of statistical data, and every technique that allows researchers to grasp and show the complexity and the relations that converge on a given object of analysis (Colombo, 2001).

This approach is being adopted, both from a theoretical and a methodological standpoint, in the frame of the transdisciplinary and multi-method project SHARE—“Seizing the Hybrid Areas of work by Re-presenting self-Employment”, with the rationale of achieving a thorough understanding of solo self-employment in six European countries: France, Germany, Italy, Slovakia, the Netherlands, and the United Kingdom. The national cases were selected on the basis of two main criteria. The first is a good balance between comparability and heterogeneity between the cases. In terms of comparability, in all the selected countries, self-employment plays a particularly important role, either because it has grown considerably over the last 20 years or because its rate is particularly high in relation to the European average. In terms of heterogeneity, these countries differ in the ways they manage the solo self-employed, and are characterized by different welfare systems. Germany and France are usually classified as conservative welfares, but with different strategies concerning women employment (Palier, 2010); the Netherlands is a hybrid case between the social-democratic and conservative models (Kammer et al., 2012); Italy represents a Southern European welfare system, with a strong reliance on family support (Ferrera, 1996); the UK is a liberal welfare state and the Slovak welfare state has shifted from a universalistic approach to a residual social system and it has recently been characterized by several employment reforms (Fenger, 2007). The second criterion is the dynamism of the cases, which has meant selecting countries where there are, or have been, documented experiences of collective actions aimed at representing solo self-employed workers, union activities, the creation of new unions and more fluid associations, with the emergence of diverse social collective actors. One of the main objectives of the project is to understand how the processes of collective organizing among the solo self-employed are connected to the spread of this category of workers, how they are culturally represented, and their level of inclusion in legal and social protection systems.

In the SHARE project, the aim is therefore, on the basis of the quantitative, qualitative and legal data collected at national level, to provide a comparative transdisciplinary analysis of how the figure of the solo self-employed differs across national contexts in terms of indicators used for their classification in national labor force surveys, employment regulations and protections, and collective representations. After this step, and on the basis of the comparative analysis, the aim is to conduct a European in-depth study on how the solo self-employed are measured, classified and represented. This means critically revising the European surveys on labor force to propose a new classification of solo self-employment; to analyse the European Union law taking into account the national legal frameworks by means of a comparison carried out with the help of cross-national ethnographic studies; and to explore the main European networks of solo self-employed workers—trade unions, associations and auto-organized networks—and to involve them in a common discussion on the data collected.

The future research agenda proposed in this contribution forms, therefore, the foundation of the ERC project SHARE, which is expected to bring a significant contribution to a more grounded understanding of the hybrid areas of work, with a particular focus on solo self-employment. The main aim is to be able, by applying the proposed transdisciplinary and multi-method approach, to construct interpretative categories able to reinvigorate the theoretical debate and challenge the old categories developed by difference with the Fordist model, such as “non-standard” or “a-typical.” Indeed, the general agreement in the scientific debate on the erosion of “standard” work arrangements has not been enough to construct new conceptual categories and challenge the binary opposition between standard and non-standard, typical and a-typical, resulting in a contrast between “the One and the Other” (Derrida, 1967). In fact, although criticized by many, the current definitions are still anchored in the categories created *ad hoc* to interpret the Fordist model. In our view, however, to define the emerging hybrid areas of work, it is not sufficient to add or subtract some properties related to traditional employment categories, since the emerging work arrangements have specific distinguishing characteristics, and the criteria to identify them have completely changed. It is for this reason that they require original theoretical lenses and research techniques, which can be built by setting a research agenda based on collective transdisciplinary and multi-method approaches.

## Data Availability Statement

Publicly available datasets were analyzed in this study. This data can be found here Eurostat (2018).

## Ethics Statement

The studies involving human participants were reviewed and approved by The research project SHARE has been reviewed and given a favorable opinion from:

- The ERC ethical review board on 22/02/2017- Ref. Ares(2017)960489;
- The Research Ethics Committee of the University of Leeds on 13/09/2017, ethics reference LTLUBS-175;
- The Research Ethics Committee of the University of Milan on 08/11/2018, ethics reference No. 50/18;
- The Data Protection Officer of the University of Milan on 10/11/2018. The patients/participants provided their written informed consent to participate in this study.

The ERC ethics officer approved all the new elements submitted for ethics on 28/05/2019.

## Author Contributions

This paper is an entirely collaborative effort by the all authors. If, however, for academic reasons individual responsibility is to be assigned. AM: wrote introduction and our proposal for a future research agenda. RB: trends and heterogeneity of solo self-employment. PD: the self-employment in the frame of labor law. PM, MM-N, and PB: the collective representation of solo self-employed workers.

### Conflict of Interest

The authors declare that the research was conducted in the absence of any commercial or financial relationships that could be construed as a potential conflict of interest.
